# M. Arthur Louis Alphonse Charpentier

**Published:** 1899-09

**Authors:** 


M. Arthur Louis Alphonse Charpentier, died at Paris, May 29, 1899. He was a Fellow of the Faculty of Medicine of Paris; member of the Academy of Medicine; chevalier of the Legion of Honor, and an Honorary Fellow of the American Association of Obstetricians and Gynecologists.

